# Leukocyte Extracellular Vesicles Predict Progression of Systolic Dysfunction in Heart Failure with Mildly Reduced Ejection Fraction (LYCHEE) – A Prospective, Multicentre Cohort Study

**DOI:** 10.1007/s12265-024-10561-3

**Published:** 2024-09-24

**Authors:** Aleksandra Gąsecka, Aleksander Siniarski, Piotr Duchnowski, Konrad Stępień, Ewelina Błażejowska, Magdalena Gajewska, Kacper Karaban, Kinga Porębska, Aleksandra Reda, Sylwester Rogula, Bartosz Rolek, Dorota Słupik, Roksana Gozdowska, Marcin Kleibert, Dominika Zajkowska, Michał Grąt, Marcin Grabowski, Krzysztof J. Filipiak, Edwin van der Pol, Rienk Nieuwland

**Affiliations:** 1 1St Chair and Department of Cardiology, Medical University of Warsaw, Banacha 1a, 02-097 Warsaw, Poland; 2 Laboratory of Experimental Clinical Chemistry & Amsterdam Vesicle Center, Amsterdam UMC, Amsterdam, The Netherlands; 3 Department of Coronary Artery Disease and Heart Failure, Institute of Cardiology, Faculty of Medicine, Jagiellonian University Medical College, Krakow, Poland; 4 St. John Paul II Hospital in Krakow, Krakow, Poland; 5 Ambulatory Care Unit, Cardinal Wyszynski National Institute of Cardiology, Warsaw, Poland; 6 Department of Thromboembolic Disorders, Institute of Cardiology, Jagiellonian University Medical College, Krakow, Poland; 7 Department of General, Gastroenterological and Oncological Surgery, Medical Universityof Warsaw, Warsaw, Poland; 8 Department of Hypertensiology, Angiology and Internal Medicine, Poznan University of Medical Sciences, Poznan, Poland; 9 Department of Clinical Sciences, Maria Sklodowska-Curie Medical Academy, Warsaw, Poland; 10 Department of Biomedical Engineering and Physics, Amsterdam UMC, Amsterdam, The Netherlands

**Keywords:** Extracellular vesicles, EVs, Heart failure, HFmrEF, Risk stratification

## Abstract

Risk stratification in heart failure with mildly-reduced ejection fraction (HFmrEF) remains challenging. We evaluated the predictive value of advanced glycation end products (AGEs) and plasma concentrations of extracellular vesicles (EVs) for the systolic and diastolic dysfunction progression in HFmrEF patients. Skin AGE accumulation was measured using AGE Reader. Plasma EV concentrations were measured using flow cytometry. Among 74 patients enrolled, 13 (18%) had systolic dysfunction progression and 5 (7%) had diastolic dysfunction progression during 6.5 months follow-up. Leukocyte EVs concentrations were higher in patients with systolic dysfunction progression (*p* = 0.002) and predicted the progression with 75.0% sensitivity and 58.3% specificity, independent of other clinical variables (OR 4.72, 95% CI 0.99–22.31). Skin AGE levels and concentrations of other EV subtypes were not associated with systolic or diastolic dysfunction progression. Increased leukocyte EVs concentrations are associated with 4.7-fold higher odds of systolic dysfunction progression in HFmrEF patients.

**Graphical Abstract:**

AGE – advanced glycation end products, HFmrEF – heart failure with mildly reduced ejection fraction, ECHO – echocardiography, EV – extracellular vesicles

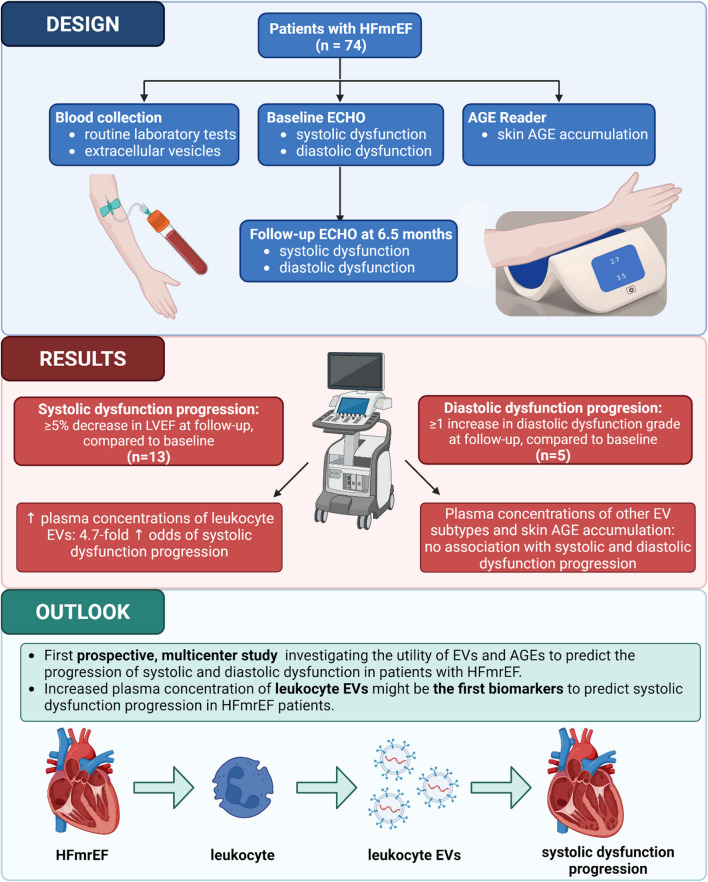

**Supplementary Information:**

The online version contains supplementary material available at 10.1007/s12265-024-10561-3.

## Introduction

Heart failure (HF) is a clinical manifestation of myocardial dysfunction resulting in a decrease in cardiac output relative to the metabolic demand of the tissues, or a compensatory increase in filling pressure to maintain cardiac output. [1] The latest 2021 European Guidelines on Heart Failure divide HF into three phenotypes: heart failure with reduced ejection fraction (HFrEF, EF < 40%), heart failure with preserved ejection fraction (HFpEF, EF > 50%) and heart failure with mildly reduced ejection fraction (HFmrEF, EF 40–49%). [1] Since HFmrEF represents a “grey zone” between the other HF phenotypes, the optimal pharmacotherapy to improve prognosis for HFmrEF patients remains challenging. All currently available drugs have a class of recommendation IIb *(“might be considered”*), except for SGLT2-inhibitors that have recently been upgraded to a class of recommendation I *(“should be administered”).* In contrast, the guideline-recommended pharmacotherapy in HFrEF patient is clear, with a class of recommendation I for four classes of drug with a proven benefit in terms of survival. [1] However, these drugs are not routinely recommended in HFmrEF, because it is not clear which patients will experience the progression of systolic dysfunction, i.e. a decrease in left ventricle ejection fraction (LVEF). Thus, novel tools to predict HFmrEF trajectory are required to stratify risk and inform treatment decisions, potentially preventing the decrease in LVEF.

Advanced glycation end products (AGEs) and extracellular vesicles (EVs) are among the novel pathophysiological factors involved in the development and progression of HF. Chronic inflammatory state in patients with HF, as reflected by increased concentrations of numerous inflammatory biomarkers (interleukin-6, tumor necrosis factor-α, CRP) leads to both increased AGE formation and increased EV release from activated red blood cells, leukocytes and platelets [2].

AGEs are a diverse group of compounds arising from the non-enzymatic reaction between reducing sugars and molecules containing an amine group, such as proteins, lipids, or nucleic acids. Increased production and accumulation of AGE in human body has been associated with aging, oxidative stress, inflammation and development of many chronic diseases. [3, 4] AGEs exert negative effects on the cardiovascular system and may aggravate both the systolic and diastolic dysfunction of the heart. Interaction of AGEs with myocardial sarcoendoplasmic reticulum Ca^2+^-ATPase 2a (SERCA2a) protein negatively affects calcium metabolism in cardiomyocytes, leading to their reduced susceptibility to stretch and subsequently to diastolic dysfunction. [5, 6] Binding of AGEs with the receptor for AGEs (RAGE) promotes atherosclerosis, which is the main cause of myocardial ischemia and systolic dysfunction [7].

Extracellular vesicles (EVs) are nanosized particles released from cells into body fluids, including blood, which serve as mediators of intercellular communication, allowing cells to exchange proteins, lipids and genetic material. [8] EVs facilitate communication between cardiomyocytes, fibroblasts, endothelial cells and the extracellular matrix thus contributing to myocardial remodeling, exerting either protective or deleterious effects on cardiovascular system, depending on their subtype and pathophysiological situation [9, 10]. The profile of plasma EVs differs in patients with HFrEF and HFpEF [11], suggesting that EVs might be potentially useful biomarkers to predict the progression of systolic and diastolic dysfunction in HFmrEF patients.

The utility of AGEs and EVs to predict outcomes in HFmrEF patients has not hitherto been studied. We hypothesized that the baseline skin levels of AGE and plasma EV concentrations are increased in patients with HFmrEF and progression of systolic or diastolic dysfunction, compared to patients without progression. The objective of this study was to evaluate the predictive value of AGEs and EVs for systolic and diastolic dysfunction progression of in patients with HFmrEF.

## Methods

### Study Design

We conducted a prospective, multicenter cohort study at 3 academic centers in Poland between February 2020 and August 2023, in collaboration with Amsterdam Vesicle Center, Amsterdam University Medical Centers (UMC), the Netherlands. The study protocol was approved by the Biomedical Ethical Committee of Medical University of Warsaw (approval number: KB/89/2019). Study population included adult, stable patients with HFmrEF, defined as LVEF 40–49% and an increased N-terminal pro B natriuretic peptide (NTproBNP) concentration (> 125 pg/ml in patients without atrial fibrillation [AF] and > 300 pg/ml in patients with AF). Written informed consent to participate in the study was obtained from each patient.

Exclusion criteria were any symptoms or signs of HF exacerbation, acute coronary syndrome or acute heart failure within the past 3 months, post-heart transplantation status, active endocarditis, pericarditis or myocarditis, advanced chronic kidney disease (estimated glomerular filtration rate < 30 ml/min), active malignancy, active autoimmune disease and skin abnormalities of the forearms that may interfere with AGE measurement.

During the index hospitalization, clinical and echocardiographic data were collected, AGE skin accumulation was measured and blood collection for laboratory tests and plasma EV concentration was performed. Control echocardiography to evaluate the progression of systolic and diastolic dysfunction compared to baseline was performed at the follow-up visit, scheduled after 6 ± 3 months.

Systolic function was measured using the biplane modified Simpson method, which requires area tracings of the left ventricle (LV) cavity in the apical 2-chamber and 4-chamber view during systole and diastole and subsequently calculates LVEF from the entire volume of the LV. Systolic dysfunction was defined as LVEF < 40%, and the progression of left ventricle (LV) systolic dysfunction was defined as a ≥ 5% decrease in LVEF at follow-up, compared to baseline. Diastolic dysfunction was defined according to the currently recommended algorithm, based on (i) mitral inflow velocity in early diastole (E wave) and mitral inflow velocity in late diastole caused by atrial contraction (A wave) (E/A ratio), (ii) average early peak wave velocity (e’) of the mitral annulus (E/e’ ratio), (iii) tricuspid regurgitation velocity and peak gradient (TRV/ TRPG) and (iv) left atrium volume index (LAVI) [12]. E/A ratio and TRV were measured using continuous-wave Doppler, average e’– using tissue Doppler imaging and LAVI – using two-dimensional echocardiography. Based on the E/A ratio, E/e’ ratio, TRV/ TRPG and LAVI, diastolic dysfunction was divided into grade I, II or III [12]. Progression of LV diastolic dysfunction was defined as a ≥ 1 increase in diastolic dysfunction grade at follow-up, compared to baseline.

Skin AGE accumulation was measured using AGE Reader, which is a non-invasive device that uses ultraviolet light to excite autofluorescence in the skin of the forearm, as previously described. [13] The AGE Reader contains an ultraviolet A lamp that emits light with a peak wavelength between 360 and 370 nm. The light reflected from the skin and emitted in the 300 to 600 nm range is measured by an in-built spectrometer using an UV glass fiber. The result is an average of 3 measurements taken immediately after each other. Skin AGE accumulation was measured only in 1 study site (Medical University of Warsaw) due to the availability of the device.

Blood collection for EV measurements were done by trained professionals according to recent guidelines to study EVs. [14] Briefly, blood was collected once into 7.5 mL 0.109 mol/L ethylenediaminetetraacetic acid (EDTA) plastic tubes (S-Monovette, Sarstedt) via antecubital vein puncture. Following preparation of platelet-depleted plasma with double centrifugation (2500 g, 15 min, 20 °C, acceleration speed 1, no brake), samples were stored at − 80 °C until analyzed, according to the recommendations to store biological samples for EV measurements. [14] Prior to analysis, samples were thawed for 1 min in a water bath (37 °C) to avoid cryoprecipitation.

Flow cytometry (A60-Micro, Apogee Flow Systems) was used to determine the concentration of EVs derived from erythrocytes (CD235a^+^), leucocytes (CD45^+^), platelets (CD61^+^), and exposing phosphatidylserine (PS^+^) in platelet-depleted plasma. To improve the reproducibility of our EV flow cytometry experiments, we (i) applied the framework for standardized reporting of EV flow cytometry experiments (MIFlowCyt-EV) [15], (ii) calibrated all detectors, (iii) and applied custom-built software to fully automate data calibration and processing. All details regarding sample collection and handling, assay controls, instrument calibration, data acquisition, and EV characterization are included in the Supplementary File.

The primary endpoint was the comparison of plasma EV concentrations in patients with and without the progression of LV systolic dysfunction. The secondary endpoints were the comparisons of (i) plasma EV concentrations in patients with and without the progression of LV diastolic dysfunction, (ii) skin AGE accumulation in patients with and without the progression of LV systolic and diastolic dysfunction.

### Statistical Analysis

As there is no data regarding the differences in skin AGE accumulation and plasma EV concentrations depending on the progression of systolic and diastolic dysfunction [7] the power calculation for the primary end-point was based on the differences in EV concentrations in patients with acute HF and healthy controls. [2] Patients with acute HF had on average twofold higher leukocyte (specifically macrophage) EV concentrations compared to controls. The required sample size was calculated by a two-sided t-test at a significance level of 0.05 with the following assumptions: (i) mean difference between the groups with and without progression of systolic dysfunction = 1.0, (ii) standard deviation (SD) ± 1.0, and (iii) nominal test power = 0.8. Hence, at least 17 patients with HFmrEF should be enrolled in the study, who will have the progression of systolic dysfunction. Considering the expected progression rate of 25% and the loss-to-follow-up rate of 5%, at least 72 patients should be enrolled.

Statistical analyses were conducted using IBM SPSS Statistics, version 27.0 (IBM, New. York, USA). Categorical variables were presented as number and percent and compared using χ2 test. The Shapiro–Wilk test was used to assess normal distribution of continuous variables. Continuous variables were presented as mean with standard deviation (SD) or median with interquartile range (IQR). Unpaired t-test or U-Mann Whitney test was used to assess the difference in variables between patients with and without progression of systolic and diastolic dysfunction. Spearman correlation coefficient was used to evaluate correlations between AGE and EVs and echocardiographic parameters of systolic and diastolic dysfunction. The predictive value of EVs for the progression of systolic dysfunction (primary endpoint) and the cut-offs were calculated using a receiver operating characteristic (ROC) curve. Logistic regression model incorporating EVs and clinical characteristics which predicted systolic dysfunction at *p* < 0.05 in the univariable analysis were included in the multivariable regression analysis. The results of univariable and multivariable regression analyses are reported as odds ratio (OR) and 95% confidence interval (CI). A two-sided *p*-value below 0.05 was considered significant.

## Results

Study design and flow chart are shown in Graphical Abstract. All patients attended the follow-up visit at the median follow-up time of 6.5 (6.0–9.6) months. Among 74 patients enrolled in the study, 13 (18%) had the progression of systolic dysfunction and 5 (7%) had the progression of diastolic dysfunction.

Patients who experienced systolic dysfunction progression had higher baseline level of creatinine (*p* = 0.010) and lower posterior wall diameter (*p* = 0.027), with no other differences regarding baseline, laboratory and echocardiographic parameters between the groups. Initially, the median LVEF was similar in both groups (45.0 (42.5–47.0) vs. 45.0 (44.0–48.0), *p* = 0.430). At follow-up, the median LVEF was lower in patients with progression of systolic dysfunction, compared to patients without the progression (42.0 (42.0–43.0) vs. 48.0 (45.0–49.0), *p* = 0.001). The diastolic function parameters were comparable in both groups at baseline and follow-up (Table 1).

**Table 1:** Comparison of baseline characteristics between patients who experienced progression of systolic dysfunction and those who did not during a median follow-up of 6.5 months

	Total population (*N* = 74)	No progression of systolic dysfunction (*N* = 61)	Progression of systolic dysfunction (*N* = 13)	*p*-value
**Baseline characteristics**				
Age, years	70.0 (63.0–78.0)	69.5 (62.0–77.5)	75 (66.0–81.0)	0.629
Gender, male	59 (79.7%)	50. (81.0%)	9 (69.2%)	0.308
BMI, kg/m2	27.6 ± 4.0	27.9 ± 3.9	26.2 ± 4.3	0.191
HF ischemic etiology	56 (75.7%)	47 (77.0%)	9 (69.2%)	0.551
**Co-morbidities**				
Hypertension	62 (84.0%)	51 (83.6%)	11 (84.6%)	0.938
Dyslipidemia	49 (66.2%)	40 (65.6%)	9 (69.2%)	0.808
Diabetes	28 (37.8%)	26 (42.6%)	2 (15.4%)	0.113
Obesity (BMI > 30 kg/m^2^)	20 (27.0%)	16 (26.2%)	4 (30.8%)	0.747
Metabolic syndrome	32 (43.2%)	27 (44.3%)	5 (38.5%)	0.710
NYHA class	2 (2–2)	2 (2–2)	2 (2–2)	0.757
**Laboratory data**				
Cholesterol, mg/dL	141.5 (121.3–179.2)	138.5 (120.5–182.7)	145.7 (131.0–164.0)	0.803
HDL, mg/dL	48.0 (38.3–56.0)	46.7 (38.0–56.5)	53.5 (45.5–54.1)	0.450
LDL, mg/dL	74.0 (57.6–114.0)	73.6 (38.0–56.5)	82.9 (66.0–90.0)	0.504
TG, mg/dL	104.5 (80.0–152.0)	105.0 (80.0–152.0)	103.6 (84.4–136.0)	0.696
Creatinine, mg/dL	1.0 (0.9–1.2)	1.1 (0.9–1.2)	0.9 (0.8–1.0)	**0.010**
eGFR, mL/min/1.73 m^2^	63.0 ± 20.0	60.0 ± 19.7	77.0 ± 19.5	0.092
NT-proBNP, pg/mL	694.0 (308–1415)	704.5 (291.0–1419.0)	694.0 (542.0–1036)	0.688
CRP, mg/L	1.5 (1.0–6.2)	1.5 (1.0–6.8)	1.40 (0.6–4.6)	0.618
RBC, *10^6^/µL	4.4 ± 0.6	4.5 ± 0.6	4.4 ± 0.6	0.644
WBC, *10^3^/µL	7.4 (6.1–8.5)	7.6 (6.2–8.5)	6.5 (4.6–8.5)	0.228
PLT, *10^3^/µL	181 (161–230)	181 (161–232)	188 (160–213)	0.930
**Baseline echocardiography**				
LVEF, %	45.0 (43.0–47.0)	45.0 (42.5–47.0)	45.0 (44.0–48.0)	0.430
LAV, ml	93.0 (52.9–134.0)	95.4 (61.0–134.0)	91.8 (43.0–69.0)	0.211
LAVI, ml/m^2^	50.1 (33.7–71.2	50.8 (32.6–71.6)	50.0 (30.2–84.5)	0.934
E wave, m/s	0.8 (0.7–0.9)	0.8 (0.6–0.9)	0.9 (0.5–1.0)	0.297
A wave, m/s	0.8 ± 0.3	0.8 ± 0.3	0.6 ± 0.2	0.260
E/A	0.9 (0.8–1.4)	0.9 (0.8–1.3)	1.1 (0.9–1.4)	0.150
DecT, ms	173.0 (146.0–222.0)	173.0 (146.0–222.0)	158.5 (136.0–353.0)	0.821
e’med, cm/s	6.6 (5.6–7.9)	6.7 (5.4–7.8)	6.4 (6.0–8.2)	0.563
e’lat, cm/s	9.3 ± 2.9	9.3 ± 3.1	9.0 ± 3.7	0.964
E/e’ average	10.1 ± 4.6	9.7 ± 4.7	11.4 ± 4.6	0.387
TRPG, mmHg	26.0 (19.0–32.0)	25.0 (19.0–32.0)	27.5 (23.5–33.5)	0.617
LVEDd, mm	54.5 (50.0–57.0)	55.0 (50.0–58.0)	52.0 (48.0–56.0)	0.206
LVEDV, mL	135.9 ± 34.0	137.3 ± 34.9	129.1 ± 45.2	0.594
LVESV, mL	71.5 (48.0–84.0)	71.5 (42.5–47.0)	58.0 (32.0–84.0)	0.673
IVSd, mm	11.0 (10.0–12.0)	11.0 (10.0–12.0)	11.0 (9.0–12.0)	0.567
PWd, mm	10.0 (9.0–11.0)	10.0 (9.0–11.0)	9.0 (8.0–10.0)	**0.027**
**Pharmacotherapy at discharge**				
Beta-blockers	68 (91.9%)	55 (90.2%)	13 (100%)	0.238
RAAS inhibitor	68 (91.9%)	55 (90.2%)	13 (100%)	0.238
MRA	41 (55.4%)	32 (52.5%)	9 (69.2%)	0.269
SGLT2-inhibitor	23 (31.1%)	19 (31.1%)	4 (30.8%)	0.979
Diuretic	42 (56.8%)	37 (60.7%)	5 (38.5%)	0.143
Statin	65 (87.8%)	54 (88.5%)	11 (84.6%)	0.695
**Echocardiography at follow-up**				
LVEF, %	46.5 (42.0–49.0)	48.0 (45.0–49.0)	42.0 (42.0–43.0)	**0.001**
LAVI, ml/m^2^	46.1 (28.0–52.4)	43.5 (28.2–53.1)	46.6 (21.5–47.6)	0.837
E wave, m/s	0.7 (0.6–1.0)	0.8 (0.6–1.0)	0.5 (0.5–1.0)	0.107
A wave, m/s	0.8 (0.6–0.9)	0.8 (0.7–0.9)	0.7 (0.4–0.8)	0.267
E/A	0.9 (0.7–1.3)	1.0 (0.7–1.3)	0.8 (0.6–0.9)	0.051
DecT, ms	183.6 ± 73.5	180.2 ± 66.7	199.1 ± 102.8	0.517
e’med, cm/s	6.0 (5.1–7.7)	6.2 (5.1–8.0)	6.0 (5.0–7.6)	0.764
e’lat, cm/s	9.1 ± 3.4	9.1 ± 3.4	9.0 ± 3.7	0.913
E/e’ average	9.3 (6.7–13.0)	10.1 (8.0–13.4)	7.3 (6.3–8.8)	0.084
TRPG, mmHg	22.0 (19.0–30.0)	22.0 (19.0–30.0)	22.0 (19.0–28.5)	0.848
LVEDd, mm	52.0 (47.0–58.0)	55.0 (47.0–59.5)	51.0 (47.5–51.5)	0.147
LVEDV, mL	141.7 ± 45.9	142.4 ± 45.0	139.0 ± 52.0	0.845
LVESV, mL	74.9 ± 27.7	71.3 ± 24.4	94.2 ± 38.4	0.121
IVSd, mm	10.0 (9.0–12.0)	11.0 (10.0–12.0)	10.0 (9.0–10.5)	0.073
PWd, mm	9.0 (8.0–11.0)	10.0 (8.0–11.0)	9.0 (7.5–9.5)	0.131

Bold *p*-values indicates significantly different (< 0.05). Data are shown as number (percentage), median (interquartile range) or mean ± standard deviation. *BMI* body mass index, *CRP* C-reactive protein, *DecT* deaceleration time, *eGFR* estimated glomerular filtration rate, *E/A ratio* transmitral peak flow velocity in early diastole (E wave) to peak flow velocity in late diastole caused by atrial contraction (A wave), *E/e’*’ transmitral peak flow velocity in early diastole (E wave) to average early peak wave velocity (e’) of the mitral annulus, *HF* heart failure, *HDL* high-density lipoproteins, *IVSd* interventricular septum diameter, *LAVI* left atrium volume index, *LDL* low-density lipoproteins, *LVEF* left ventricle ejection fraction, *LVEDV* left ventricle end-diastolic volume, *LVESV* left ventricle end-systole volume, *MRA* mineralocorticoid receptor antagonist, *NT-proBNP* N-terminal pro B natriuretic peptide, *NYHA* New York Heart Association, *PLT* platelets, *PWd* posterior wall diameter, *RAAS* renin–angiotensin–aldosterone system, *RBC* red blood cells, *SGLT2i* sodium-glucose cotransporter-2 inhibitors, *TG* triglycerides, *TRPG* tricuspid regurgitation peak gradient, *WBC* white blood cells

**Fig. 1:**
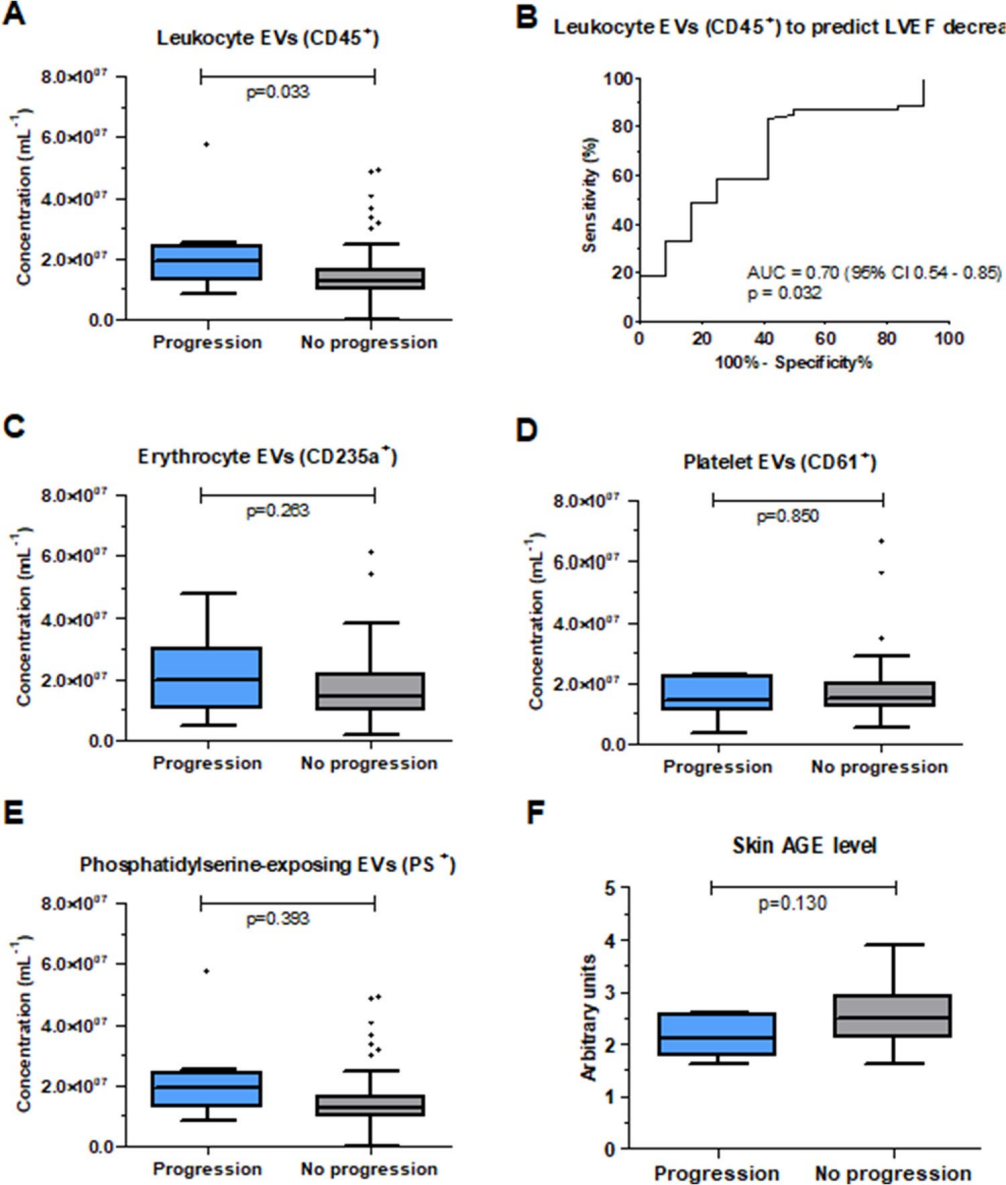
Baseline plasma concentrations of extracellular vesicles (EVs) and skin levels of advanced glycation end products (AGE) in patients who did and did not experience the progression of left ventricle systolic dysfunction at follow-up (panels A, C-F). Receiver operating curve (ROC) showing the predictive value of leukocyte EVs (CD45.^+^) for prediction of systolic dysfunction (panel B). The flow cytometer detection range was 80–10,000 nm and > 50 MESF PE for erythrocyte EVs; 150–1,000 nm and > 50 MESF PE for leukocyte EVs; 50–10,000 nm and > 50 MESF APC for platelet EVs and 500–100,000 nm and > 50 MESF APC for phosphatidylserine (PS)-exposing EV. Number of patients: 74

**Fig. 2:**
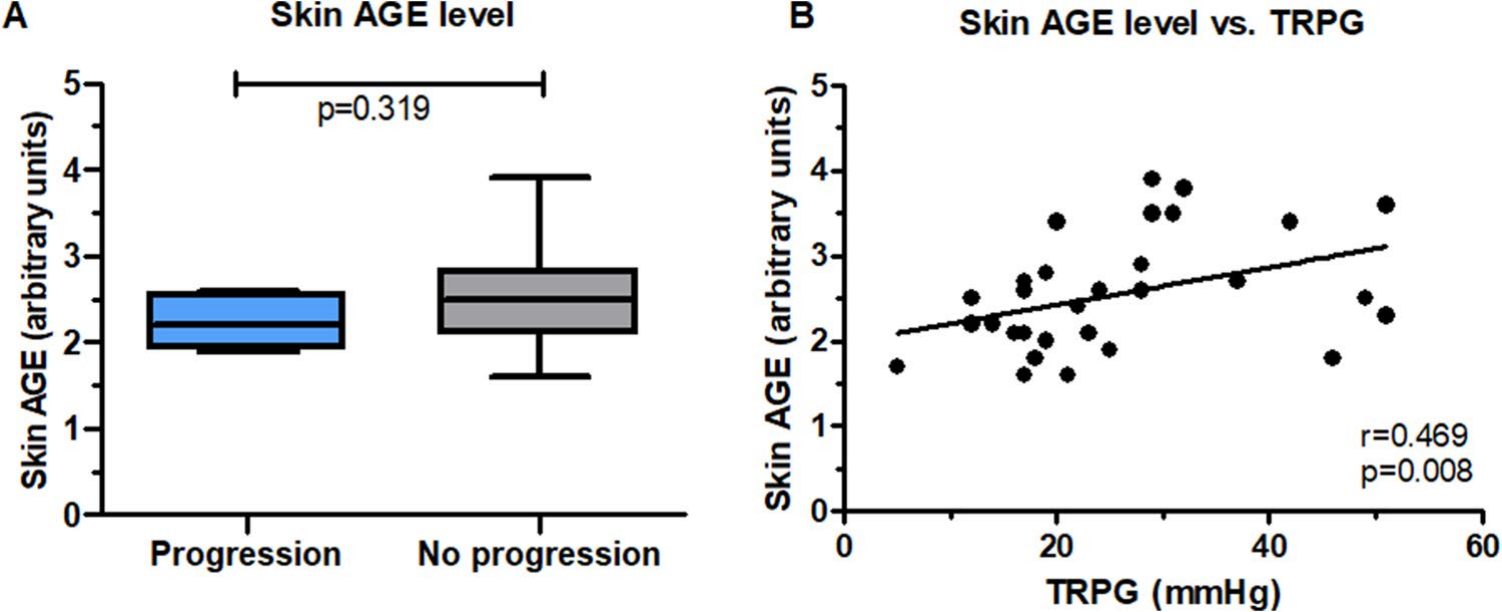
Skin levels of advanced glycation end products (AGE) in patients who did and did not experience the progression of left ventricle diastolic dysfunction at follow-up (panel A) and correlation between AGE and tricuspid peak regurgitation gradient (TRPG) at baseline (panel B). Number of patients: 40

Figure 1 shows the plasma concentrations of EVs within the detection range of the flow cytometer and AGE levels in patients with and without progression of left ventricle systolic dysfunction at follow-up. Baseline concentrations of leukocyte EVs (CD45 +) were higher in patients who experienced the progression of systolic dysfunction, compared to those who did not (*p* = 0.002; Fig. 1A) and discriminated between these two groups of patients (area under ROC curve (AUC) = 0.70, *p* = 0.032; Fig. 1B). Concentrations of other analyzed EV subtypes as well as skin AGE levels did not differ among patients with and without the progression of systolic dysfunction (Fig. 1C-2F).

Statistical estimates for prediction of systolic dysfunction by baseline plasma concentration of leukocyte EVs are shown in Table 2. The results of univariable and multivariable analysis are shown in Supplementary Table 1 and Table 3. Increased baseline concentration of leukocyte EVs above the cut-off value, defined as 1.35*10^7^ particles per mL plasma based on the ROC curve, predicted systolic dysfunction with 75.0% sensitivity and 58.3% specificity. In multivariable analysis, only baseline leukocyte EVs concentration > 1.35 *10^7^ per mL and creatinine concentration were predictive of systolic dysfunction (OR 4.72, 95% CI 0.99–22.31, *p* = 0.049 for leukocyte EVs, OR 0.03, 95% CI 0.01–0.43, *p* = 0.022 for creatinine, respectively).

**Table 2:** Statistical estimates for prediction of systolic dysfunction by baseline plasma concentration of leukocyte extracellular vesicles (EVs), based on receiver operating characteristic (ROC) analysis

	AUC (95% CI)	*p*-value	Cut-off	Sensitivity	Specificity	PPV	NPV
Leu EVs	0.70 (0.54–0.85)	0.032	1.35 *10^7^ per mL plasma	75.0%	58.3%	26.5%	92.1%

*AUC* area under the curve, *CI* confidence interval, *EVs* extracellular vesicles, *PPV* positive predictive value, *NPV* negative predictive value

**Table 3:** Results of multivariable analysis to predict left ventricle systolic dysfunction using the concentration of leukocyte extracellular vesicles above the cut-off value and clinical variables

	OR	95% CI		*p*-value
		Lower	Upper	
Leu EVs, > 1.35 *10^7^ mL^−1^	4.72	0.99	22.31	**0.049**
Age, years	1.02	0.96	1.07	0.566
Gender, male	0.39	0.05	2.92	0.356
Creatinine, mg/dL	0.03	0.01	0.43	**0.022**

*CI* confidence interval, *EVs* extracellular vesicles, *OR* odds ratio

Patients who experienced diastolic dysfunction progression had lower LVEF at baseline and follow-up (*p* = 0.049), with no other differences between the groups (Supplementary Table 2). There were no differences in EV concentrations (Supplementary Fig. 1) and AGE levels (Fig. 2A) in patients with and without the progression of diastolic dysfunction at follow-up. There was a positive correlation between skin AGE level and tricuspid regurgitation velocity and peak gradient (TRPG) (r = 0.469, *p* = 0.008; Fig. 2B). There were no significant correlations between plasma concentrations of EVs and other echocardiographic parameters of systolic and diastolic dysfunction (Supplementary Table 3).

## Discussion

To our best knowledge, this is the first prospective, multicenter study investigating the utility of novel pathophysiological factors related to the development and progression of heart failure, EVs and AGEs to predict progression of systolic and diastolic dysfunction in patients with HFmrEF. The main findings of our study are that: (i) patients with increased plasma concentrations of leukocyte EVs had nearly fivefold higher odds of systolic dysfunction progression, (ii) there was no association between plasma concentrations of other EV subtypes and the progression of systolic or diastolic dysfunction, (iii) there was no association between skin AGE accumulation and the progression of systolic or diastolic dysfunction in HFmrEF during 6-months follow-up.

Research on the concentration and function of EVs in HF patients remains inconclusive.

A study involving 119 chronic HF patients (both HFrEF and HFpEF) and 60 matched controls demonstrated that plasma concentrations of EVs from leukocytes (CD45^+^), specifically monocytes (CD16^+^), neutrophils (CD15^+^), T-lymphocytes (CD3^+^) and natural-killer cells (CD56^+^) were higher in chronic HF patients than in controls. Patients with more severe HF, based on a higher NYHA classification score, had higher levels of leukocytes EVs (CD45^+^), specifically those derived from T-lymphocytes (CD3^+^). [16] We observed higher baseline concentrations of leukocyte EVs (CD45^+^) in patients with subsequent progression of systolic dysfunction, compared to those without progression, which might reflect more severe chronic inflammatory state and/or higher severity of HF. [17] To support this notion, we found a moderate, positive correlation between leukocyte count and leukocyte EV concentration (r = 0.267, *p* = 0.048), which was not present in case of erythrocyte and erythrocyte EVs or platelets and platelet EVs (Supplementary Fig. 2). Considering that chronic HF is a persistent inflammatory state, increased leukocyte EVs might reflect either increased leukocyte EV release, less efficient clearance, or both. Increased leukocyte EV release might contribute to systolic dysfunction progression in HF patients. Leukocytes are among the first cells mobilized to the damaged myocardium following ischemia, where they promote inflammation, tissue repair and fibrosis, with the ultimate goal of wound healing. If the pro-inflammatory milieu persists without a transition to an anti-inflammatory phenotype, leukocyte infiltration leads to maladaptive remodelling, fibrosis and scar formation, exacerbating systolic dysfunction. [18, 19] Since most patients in our study presented with an ischemic (atherosclerotic) HF aetiology, higher leukocyte EV concentrations might have contributed to the disease progression. Previous reports also showed that leukocyte EV concentrations are higher in patients with subclinical atherosclerosis and associated with atherosclerotic plaque vulnerability [20, 21].

Since we (i) did not investigate leukocyte EVs directly in the myocardium, (ii) did not perform any functional test on leukocyte EV function, and (iii) did not measure EV from various leukocyte subpopulations, our results remain hypothesis-generating.

Regarding the clinical implication of our finding, the association between higher baseline concentration of leukocyte EVs and progression of systolic dysfunction in HFmrEF patients might provide a rationale for the initiation of HF pharmacotherapy (beta-blockers, angiotensin-converting-enzyme inhibitors or angiotensin receptor-neprilysin inhibitors, mineralocorticoid receptor antagonists and SGLT2-inhibitors) at an earlier stage to prevent progression to HFrEF. The first point-of-care tests using EVs as biomarkers have already been developed (e.g. ExoDx™ Prostate IntelliScore test based on urinary EVs, Exosome Diagnostics, Germany). Introduction of a similar test based on leukocyte EVs might improve risk stratification in HFmrEF patients and inform treatment decision during routine ambulatory visits.

We found no association between plasma concentrations of other EV subtypes (erythrocyte-derived, platelet-derived and exposing PS) and the progression of systolic or diastolic dysfunction. In contrast, previous studies demonstrated that even optimally treated chronic HF patients had higher plasma concentrations of PS^+^ EVs, compared to healthy controls [16, 22, 23]. Our study focused only on HF patients with and without the progression of systolic dysfunction, but did not include healthy controls, precluding a head-to-head comparison with previous studies.

One study showed higher plasma concentrations of PS^+^ EVs in acute HF following myocardial infarction, but higher plasma concentrations of PS^−^ EVs in chronic stable HF. Negatively charged phospholipids including PS exposed on EVs surface binds positively charged clotting factors, promoting coagulation [24]. PS exposure can be an artifact due to freeze-thawing of residual platelets present in plasma following most centrifugation protocols [25], so the results should be interpreted with caution. Nevertheless, the authors hypothesized that the PS exposure on EVs might distinguish between two populations with different roles, with PS^+^ EVs reflecting the procoagulant state and PS^−^ EVs reflecting the proinflammatory state. [22, 26, 27] The observed difference in leukocyte concentrations among patients with and without the progression of systolic dysfunction in our study, but no difference in PS^+^ EV concentrations might suggest the predominant role of inflammation in systolic dysfunction progression. However, since our study is the first which specifically included only patients with HFmrEF, whereas previous studies focused either on HFrEF or HFpEF patients, or did not differentiate between HF phenotypes, head-to-head comparisons between our results and results of other authors cannot be made.

Studies focusing on HFrEF reported a correlation between both elevated and reduced AGE levels and systolic dysfunction [28], or have suggested no association between AGEs and systolic dysfunction [29–31]. In line with the recent study, we also found comparable AGE levels in patients with and without the progression of systolic dysfunction, suggesting no diagnostic value of AGE for risk stratification in HFmrEF patients. In contrast, in HFpEF patients who mostly suffer from diastolic dysfunction, the association between AGEs levels and diastolic dysfunction has been consistently reported [7, 29, 30]. We found a clear positive correlation between AGE level and TRPG, which is a marker of increased pressures in the pulmonary artery, a surrogate of left atrium pressure and one of the hallmarks of diastolic dysfunction. However, we did not observe any association between skin AGE accumulation and diastolic dysfunction, nor any correlation between skin AGE accumulation and other parameters of diastolic dysfunction, except for TRPG. This could be caused by the fact that both previous studies included only subjects with diabetes and evaluated only isovolumetric relaxation time as a surrogate for impaired left ventricular relaxation, whereas the currently-recommended parameters of diastolic dysfunction (E/A ratio, E/e’ ratio, LAVI, TRPG) were not measured [29, 30]. Considering the methodological differences, whether elevated skin AGE accumulation indeed reflects diastolic dysfunction remains to be further clarified.

## Strengths and Limitations

There are both clinical and methodological strengths of this study. From a clinical perspective, this is the first prospective, multicenter study which assessed the clinical utility of AGEs and EVs to predict outcomes in HFmrEF patients. For the first time, we demonstrated that increased plasma concentration of leukocyte EVs are associated with a fivefold higher odds of systolic dysfunction progression in HFmrEF patients. From a methodological perspective, we used calibrated flow cytometry and fully transparent reporting to improve reliability and reproducibility of our results. Previously, we showed that the recommended and most commonly used centrifugation protocol to prepare cell-free plasma does not remove all platelets, and that the presence of residual platelets affects downstream analysis [25]. Following a freeze–thaw cycle, fragmented residual platelets may unspecifically bind to monoclonal antibodies, impacting the concentrations of various EV subtypes, including EVs from leukocytes. In our study, platelet EV concentrations after a freeze–thaw cycle were comparable between patients, indicating that blood collection and plasma preparation were well-controlled.

There are also limitations of this study, which should be acknowledged. First, the study group was relatively small (*n* = 74) and the follow-up time short (median 6.5 months), highlighting the need for further research with larger cohorts and longer observation. Second, there was no core laboratory to evaluate echocardiographic data, which might have introduced bias due to the inter-observer variability. Third, we did not collect data regarding the compliance to the recommended pharmacotherapy, nor the changes in pharmacotherapy during the follow-up time, which might have affected the progression of LVEF systolic dysfunction. Fourth, previous studies showed that leukocyte EVs are elevated in numerous cardiovascular diseases, limiting the specificity of our finding. However, considering the current lack of biomarkers to predict HFmrEF progression, this limitation does not exclude the diagnostic utility of leukocyte EVs in HFmrEF patients. Fifth, plasma EV concentrations are affected by numerous patients’ characteristics, including age, gender, comorbidities and administered pharmacotherapy. We addressed these potential confounders in the in the univariable and multivariable analysis, ultimately showing that only leukocyte EV concentration and creatinine are independent predictors of systolic dysfunction progression. However, factors not included in the analysis might have also affected the results, such as diet and hormonal status in women. Sixth, we did not measure EVs from different subpopulation of leukocytes (i.e. neutrophils, monocytes etc.), which might have helped to understand the mechanisms underlying increased leukocyte EV concentrations in patients with systolic dysfunction progression. Eighth, the quality of the prepared plasma was not quantified according to MIBlood-EV recommendations [32], which were published last year, when the sample collection for this study was already completed. Ninth, whereas we found that creatinine level is associated with systolic dysfunction progression in HFmrEF patients, we could not expand this finding into the relationship between urinary microalbumin level or ratio of urinary microalbumin to creatinine, because these laboratory tests are not routinely performed in cardiology departments participating in the study. Finally, skin AGE accumulation was measured only in 40 patients enrolled at the Medical University of Warsaw site due to the availability of the device, and only 5 patients in our study experienced the progression of diastolic dysfunction. Hence, the analysis of association between AGE and diastolic dysfunction is largely underpowered, hampering any firm conclusions.

## Conclusions

Patients with HFmrEF and increased plasma concentrations of leukocyte EVs have nearly fivefold higher odds of systolic dysfunction progression. The next step is to conduct a multicenter trial specifically focusing on leukocyte EVs as biomarkers of systolic dysfunction progression in HFmrEF.

## Supplementary Information

Below is the link to the electronic supplementary material.

Supplementary file1 (DOCX 267 KB)

Supplementary file2 (DOCX 326 KB)

## Data Availability

The datasets generated during and/or analysed during the current study are available from the corresponding author on reasonable request.

## Abbreviations

AGEs: Advanced glycation end products
AF: Atrial fibrillation
BMI: Body mass index
ECG: Electrocardiography
ECHO: Echocardiography
EVs: Extracellular vesicles
GFR: Glomerular filtration rate
HbA1c: Blood glycated hemoglobin
HF: Heart failure
HFmrEF: Heart failure with mildly reduced ejection fraction
HFpEF: Heart failure with preserved ejection fraction
HFrEF: Heart failure with reduced ejection fraction
MACCE: Major adverse cardiac and cerebrovascular events
NT-proBNP: N-terminal pro B natriuretic peptide
TTE: Transthoracic echocardiography
