# Supplementary material for: Leukocyte Extracellular Vesicles Predict Progression of Systolic Dysfunction in Heart Failure with Mildly Reduced Ejection Fraction (LYCHEE) – A Prospective, Multicentre Cohort Study
Source: J Cardiovasc Transl Res. 2024 Sep 24;18(1):17–27. doi: 10.1007/s12265-024-10561-3 (PMC11885366; doi:10.1007/s12265-024-10561-3)
Supplement: Supplementary file 1 — Supplementary file1 (DOCX 267 KB) [file 12265_2024_10561_MOESM1_ESM.docx]

Leukocyte extracellular vesicles predict progression of systolic dysfunction in heart failure with mildly reduced ejection fraction (LYCHEE) – a prospective, multicentre cohort study

Short title: EVs predict systolic dysfunction in HFmrEF

**Supplementary Materials**

Supplementary Table 1. Results of univariable analysis to predict left ventricle systolic dysfunction using the concentration of leukocyte extracellular vesicles above the cut-off value and clinical variables.

|  | OR | 95% CI | | p-value |
| --- | --- | --- | --- | --- |
|  |  | Lower | Upper |  |
| Leu EVs, >1.35 *10^7^ mL^-1^ | 4.20 | 1.03 | 17.09 | **0.045** |
| Age, years | 1.00 | 0.95 | 1.05 | 0.854 |
| Gender, male | 2.02 | 0.53 | 7.77 | 0.306 |
| BMI, kg/m^2^ | 0.89 | 0.76 | 1.06 | 0.191 |
| HF aetiology | 1.49 | 0.39 | 5.59 | 0.400 |
| Hypertension | 1.08 | 0.21 | 5.63 | 0.929 |
| Dyslipidemia | 1.18 | 0.33 | 4.29 | 0.800 |
| Diabetes mellitus | 0.245 | 0.050 | 1.200 | 0.083 |
| Metabolic syndrome | 0.79 | 0.23 | 2.68 | 0.702 |
| NYHA class | 0.82 | 0.31 | 2.19 | 0.690 |
| NT-proBNP, pg/mL | 1.00 | 1.00 | 1.00 | 0.962 |
| Creatinine, mg/dL | 0.19 | 0.01 | 0.50 | **0.018** |
| RBC, *10^6^/µL | 0.91 | 0.33 | 2.54 | 0.860 |
| WBC, *10^3^/µL | 0.99 | 0.73 | 1.35 | 0.974 |
| PLT, *10^3^/µL | 0.99 | 0.98 | 1.01 | 0.765 |
| LVEF, % | 1.12 | 0.93 | 1.36 | 1.120 |
| IVSd, mm | 0.96 | 0.79 | 1.15 | 0.631 |
| PWd, mm | 0.79 | 0.60 | 1.04 | 0.097 |
| E/A | 1.45 | 0.46 | 4.54 | 0.527 |
| E/e’ average | 1.07 | 0.91 | 1.28 | 0.380 |
| LAVI, ml/m^2^ | 0.97 | 0.90 | 1.03 | 0.289 |
| TRV, m/s | 1.66 | 0.90 | 3.04 | 0.102 |
| Beta-blockers | 0.99 | 0.97 | 1.02 | 0.585 |
| RAAS inhibitor | 0.99 | 0.97 | 1.02 | 0.585 |
| MRA | 2.03 | 0.57 | 7.34 | 0.275 |
| SGLT2-inhibitor | 0.98 | 0.27 | 3.59 | 0.979 |
| Diuretic | 0.41 | 0.12 | 1.37 | 0.150 |
| Statin | 0.71 | 0.13 | 3.90 | 0.696 |

BMI – body mass index, CRP – C-reactive protein, CI - confidence interval, DecT – deaceleration time, eGFR – estimated glomerular filtration rate, EVs - extracellular vesicles, E/A ratio - transmitral peak flow velocity in early diastole (E wave) to peak flow velocity in late diastole caused by atrial contraction (A wave), E/e’ - transmitral peak flow velocity in early diastole (E wave) to average early peak wave velocity (e’) of the mitral annulus, HF – heart failure, IVSd – interventricular septum diameter, LAVI - left atrium volume index, LVEF - left ventricle ejection fraction, MRA - mineralocorticoid receptor antagonist, NT-proBNP - N-terminal pro B natriuretic peptide, NYHA - New York Heart Association, OR – odds ratio, PLT – platelets, PWd – posterior wall diameter, RAAS – renin-angiotensin-aldosterone system, RBC - red blood cells, SGLT2i - sodium-glucose cotransporter-2 inhibitors, TRPG - tricuspid regurgitation peak gradient, WBC - white blood cells

Supplementary Table 2. Comparison of baseline characteristics between patients who experienced progression of diastolic dysfunction and those who did not during a median follow-up of 6.5 months.

|  | Total population  (N=74) | No progression of diastolic dysfunction  (N=69) | Progression of diastolic dysfunction (N=5) | p-value |
| --- | --- | --- | --- | --- |
| **Baseline characteristics** | | | | |
| Age, years | 70.0 (63.0-78.0) | 69.5 (63.5-78.5) | 73.0 (61.0-77.0) | 1.000 |
| Gender, male | 59 (79.7%) | 55 (80.0%) | 9 (69.2%) | 1.000 |
| BMI, kg/m2 | 27.8 (24.8-30.4) | 27.7 (24.5-30.4) | 29.0 (27.1-29.0) | 0.494 |
| HF ischemic etiology | 56 (75.7%) | 51 (73,9%) | 5 (100%) | 0.189 |
| **Co-morbidities** | | | | |
| Hypertension | 62 (84.0%) | 57 (83.6%) | 5 (100%) | 1.000 |
| Dyslipidemia | 49 (66.2%) | 40 (65.2%) | 4 (80%) | 1.000 |
| Diabetes | 28 (37.8%) | 26 (37.7%) | 2 (40.0%) | 0.918 |
| Obesity (BMI>30 kg/m^2^) | 20 (27.0%) | 18 (26.1%) | 2 (40%) | 1.000 |
| Metabolic syndrome | 32 (43.2%) | 31 (44.9%) | 1 (20%) | 1.000 |
| NYHA class | 2 (2-2) | 2 (2-2) | 2 (2-2) | 1.000 |
| **Laboratory data** | | | | |
| Cholesterol, mg/dL | 141.5 (121.3-179.2) | 140.0 (120.0-182.4) | 144.4 (130.0-158.0) | 0.587 |
| HDL, mg/dL | 48.0 (38.3-56.0) | 47.0 (38.0-49.5) | 54.1 (42.0-58.0) | 0.790 |
| LDL, mg/dL | 74.0 (57.6-114.0) | 74.0 (57.0-114.0) | 81.0 (71.0-85.2) | 0.504 |
| TG, mg/dL | 104.5 (80.0-152.0) | 104.3 (79.7-152.0) | 119.0 (102.8-147.0) | 0.399 |
| Creatinine, mg/dL | 1.0 (0.9-1.2) | 1.0 (0.9-1.2) | 1.0 (1.0-1.2) | 0.771 |
| eGFR, mL/min/1.73 m^2^ | 63.0±20.0 | 62.0 (58.0-82.0) | 71.0 (51.0-82.0) | 0.923 |
| NT-proBNP, pg/mL | 694 (308-1415) | 685 (305-1327) | 711 (327-1734) | 0.721 |
| CRP, mg/L | 1.5 (1.0-6.2) | 1.5 (1.0-5.5) | 4.35 (1.05-7.9) | 0.623 |
| RBC, *10^6^/µL | 4.4±0.6 | 140.0 (120.0-182.4) | 144.4 (130.0-158.0) | 0.587 |
| WBC, *10^3^/µL | 7.4 (6.1-8.5) | 47.0 (38.0-49.5) | 54.1 (42.0-58.0) | 0.790 |
| PLT, *10^3^/µL | 181 (161-230) | 74.0 (57.0-114.0) | 81.0 (71.0-85.2) | 0.504 |
| **Baseline echocardiography** | | | | |
| LVEF, % | 45.0 (43.0-47.0) | 45.0 (43.0-48.0) | 41.0 (41.0-45.0) | **0.049** |
| LAV, ml | 93.0 (52.9-134.0) | 82.0 (52.9-134.0) | 94.8 (93.0-96.6) | 0.890 |
| LAVI, ml/m^2^ | 50.1 (33.7-71.2 | 51.5 (30.6-74.8) | 46.0 (45-46.8) | 0.530 |
| E wave, m/s | 0.8 (0.7-0.9) | 0.8 (0.6-1.0) | 0.7 (0.7-0.7) | 0.460 |
| A wave, m/s | 0.8±0.3 | 0.8 (0.6-0.9) | 0.7 (0.5-0.9) | 0.820 |
| E/A | 0.9 (0.8-1.4) | 0.9 (0.8-1.4) | 0.9 (0.9-1.1) | 1.000 |
| DecT, ms | 173.0 (146.0-222.0) | 173.0 (151.0-222.0) | 140.5 (124.0-229.0) | 0.250 |
| e’med, cm/s | 6.6 (5.6-7.9) | 6.6 (5.6-7.8) | 6.3 (6.0-8.0) | 0.868 |
| e’lat, cm/s | 9.3±2.9 | 8.5 (7.1-11.7) | 10.0 (8.5-11.2) | 0.517 |
| E/e’ average | 10.1±4.6 | 10 (8.0-13.0) | 30.5 (21.5-35.5) | 0.531 |
| TRPG, mmHg | 26.0 (19.0-32.0) | 25.0 (19.0-32.0) | 30.5 (21.5-35.5) | 0.673 |
| LVEDd, mm | 54.5 (50.0-57.0) | 55.0 (50.0-57.0) | 52.0 (51.0-55.0) | 0.656 |
| LVEDV, mL | 135.9±34.0 | 132.0 (116.5-153.0) | 137.0 (120.0-142.0) | 0.981 |
| LVESV, mL | 71.5 (48.0-84.0) | 67.0 (48.0-83.0) | 96.0 (96.0-96.0) | 1.000 |
| IVSd, mm | 11.0 (10.0-12.0) | 11.0 (10.0-12.0) | 12.0 (12.0-13.0) | 0.222 |
| PWd, mm | 10.0 (9.0-11.0) | 10.0 (9.0-11.0) | 11.0 (11.0-12.0) | 0.127 |
| **Pharmacotherapy at discharge** | | | | |
| Beta-blockers | 68 (91.9%) | 63 (91.3%) | 5 (100.0%) | 0.492 |
| RAAS inhibitor | 68 (91.9%) | 63 (91.3%) | 5 (100.0%) | 0.492 |
| MRA | 41 (55.4%) | 37 (53.6%) | 4 (80.0%) | 0.252 |
| SGLT2-inhibitor | 23 (31.1%) | 21 (30.4%) | 2 (40.0%) | 0.655 |
| Diuretic | 42 (56.8%) | 39 (56.5%) | 3 (60.0%) | 0.880 |
| Statin | 65 (87.8%) | 60 (86.9%) | 5 (100.0%) | 0.389 |
| **Echocardiography at follow-up** | | | | |
| LVEF, % | 46.5 (42.0-49.0) | 47.0 (42.0-49.0) | 45.0 (42.0-45.0) | **0.049** |
| LAVI, ml/m^2^ | 46.1 (28.0-52.4) | 46.1 (28.4-52.4) | 35.1 (21.5-51.8) | 0.697 |
| E wave, m/s | 0.7 (0.6-1.0) | 0.7 (0.6-1.0) | 0.7 (0.5-0.9) | 0.729 |
| A wave, m/s | 0.8 (0.6-0.9) | 0.8 (0.6-0.9) | 0.7 (0.6-0.8) | 0.330 |
| E/A | 0.9 (0.7-1.3) | 0.9 (0.6-0.9) | 1.0 (0.7-1.3) | 0.757 |
| DecT, ms | 190.0 (114.5-230.0) | 188.5 (121.5-235.5) | 201.5 (135.5-212.5) | 0.951 |
| e’med, cm/s | 6.0 (5.1-7.7) | 6.0 (5.1-7.9) | 6.0 (5.8-6.4) | 0.934 |
| e’lat, cm/s | 9.1±3.4 | 8.9 (6.4-11.2) | 10.0 (9.6-10.9) | 0.521 |
| E/e’ average | 9.3 (6.7-13.0) | 9.4 (6.7-13.4) | 9.3 (8.0-11.0) | 0.782 |
| TRPG, mmHg | 22.0 (19.0-30.0) | 22.0 (19.0-30.0) | 20.0 (19.0-37.0) | 1.000 |
| LVEDd, mm | 52.0 (47.0-58.0) | 52.0 (47.0-58.0) | 51.0 (48.0-60.0) | 0.874 |
| LVEDV, mL | 141.7±45.9 | 145.0 (111.0-175.0) | 68.9 (45.8-92.0) | 0.900 |
| LVESV, mL | 74.9±27.7 | 77.0 (55.0-97.0) | 68.9 (45.8-92.0) | 0.698 |
| IVSd, mm | 10.0 (9.0-12.0) | 10.0 (9.0-12.0) | 11.0 (10.0-11.0) | 1.000 |
| PWd, mm | 9.0 (8.0-11.0) | 9.0 (8.0-10.5) | 9.0 (8.0-11.0) | 0.841 |

Bold p-values indicates significantly different (< 0.05). Data are shown as number (percentage), median (interquartile range) or mean +/- standard deviation. BMI – body mass index, CRP – C-reactive protein, DecT – deaceleration time, eGFR – estimated glomerular filtration rate, E/A ratio - transmitral peak flow velocity in early diastole (E wave) to peak flow velocity in late diastole caused by atrial contraction (A wave), E/e’ - transmitral peak flow velocity in early diastole (E wave) to average early peak wave velocity (e’) of the mitral annulus, HF – heart failure, HDL – high-density lipoproteins, IVSd – interventricular septum diameter, LAVI - left atrium volume index, LDL – low-density lipoproteins, LVEF - left ventricle ejection fraction, LVEDV – left ventricle end-diastolic volume, LVESV – left ventricle end-systole volume, MRA - mineralocorticoid receptor antagonist, NT-proBNP - N-terminal pro B natriuretic peptide, NYHA - New York Heart Association, PLT – platelets, PWd – posterior wall diameter, RAAS – renin-angiotensin-aldosterone system, RBC - red blood cells, SGLT2i - sodium-glucose cotransporter-2 inhibitors, TG – triglycerides, TRPG - tricuspid regurgitation peak gradient, WBC - white blood cells

Supplementary Table 3. Correlations between baseline plasma concentrations of extracellular vesicles (EVs) and AGE skin accumulation and echocardiographic parameters of systolic and diastolic dysfunction. Significant correlations are made bold and marked with a star. * p<0.05, **p<0.01, ***p<0.001.

| Marker | LVEF | E/A ratio | E/e’ ratio | LAVI | TRPG |
| --- | --- | --- | --- | --- | --- |
| Leukocyte EVs | 0.011 | -0.038 | 0.081 | -0.035 | 0.149 |
| Erythrocyte EVs | 0.019 | -0.062 | -0.163 | -0.048 | 0.021 |
| Platelet EVs | -0.047 | -0.158 | -0.213 | -0.80 | -0.015 |
| PS-exposing EVs | -0.121 | -0.025 | -0.109 | -0.061 | -0.121 |
| Skin AGE | 0.004 | 0.378 | 0.124 | 0.018 | **0.469**** |

E/A ratio - transmitral peak flow velocity in early diastole (E wave) to peak flow velocity in late diastole caused by atrial contraction (A wave), E/e’ - transmitral peak flow velocity in early diastole (E wave) to average early peak wave velocity (e’) of the mitral annulus, LAVI - left atrium volume index, LVEF - left ventricle ejection fraction, TRPG - tricuspid regurgitation peak gradient

Supplementary Figure 1. Baseline plasma concentrations of extracellular vesicles (EVs) in patients who did and did not experience the progression of left ventricle diastolic dysfunction at follow-up (panels A-D). The flow cytometer detection range was 80-10,000 nm and >50 MESF PE for erythrocyte EVs; 150-1,000 nm and >50 MESF PE for leukocyte EVs; 50-10,000 nm and >50 MESF APC for platelet EVs and 500-100,000 nm and >50 MESF APC for phosphatidylserine (PS)-exposing EV. Number of patients: 74


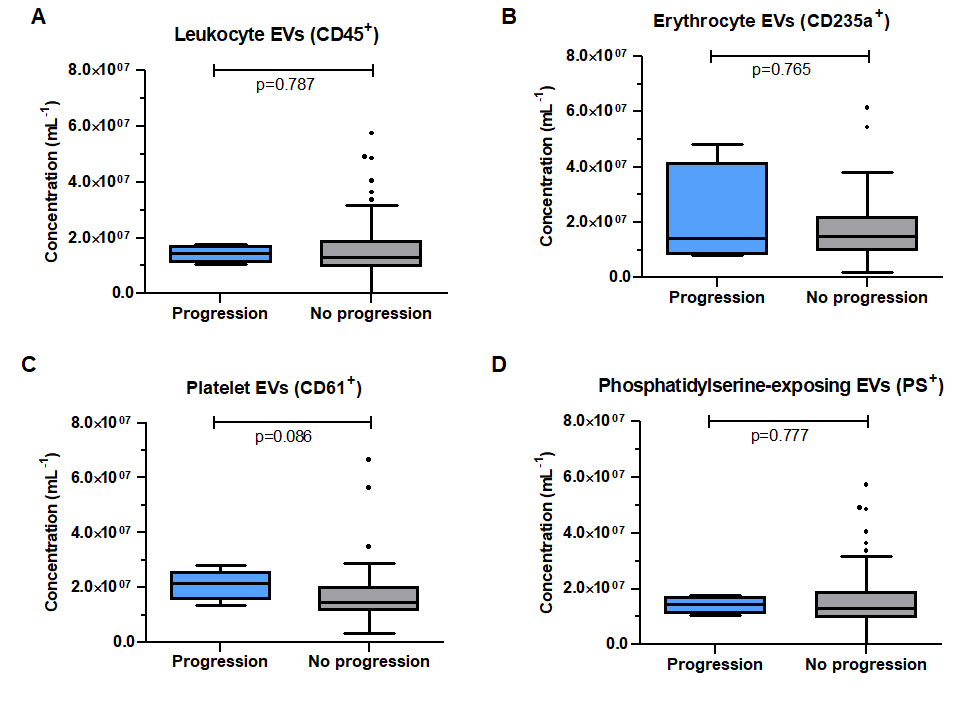


Supplementary Figure 2. Correlations between blood counts and blood count-derived extracellular vesicles (EVs). Number of patients: 74

**
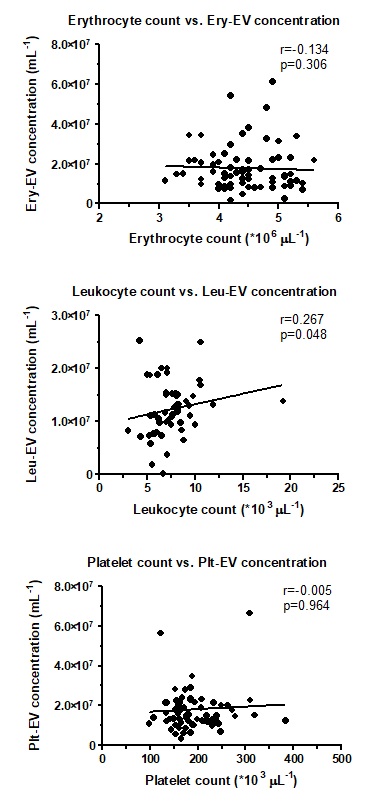
**
